# Genetic Variations of *CD40* and *LTβR* Genes Are Associated With Increased Susceptibility and Clinical Outcome of Non-Small-Cell Carcinoma Patients

**DOI:** 10.3389/fonc.2021.721577

**Published:** 2021-09-17

**Authors:** Foteinos-Ioannis D. Dimitrakopoulos, Anna G. Antonacopoulou, Anastasia E. Kottorou, Melpomeni Kalofonou, Nikolaos Panagopoulos, Dimitrios Dougenis, Thomas Makatsoris, Vasiliki Tzelepi, Angelos Koutras, Haralabos P. Kalofonos

**Affiliations:** ^1^Division of Oncology, Department of Medicine, University of Patras, Patras, Greece; ^2^Molecular Oncology Laboratory, Division of Oncology, Department of Medicine, University of Patras, Patras, Greece; ^3^Centre for Bio-Inspired Technology, Institute of Biomedical Engineering, Imperial College London, London, United Kingdom; ^4^Department of Cardiothoracic Surgery, University of Patras, Patras, Greece; ^5^Department of Pathology, University of Patras, Patras, Greece

**Keywords:** SNP, NSCLC, rs1883832, rs10849448, rs7290134, prognosis, risk

## Abstract

**Background:**

Immune system-related receptors CD40 (tumor necrosis factor receptor superfamily member 5), BAFFR (tumor necrosis factor receptor superfamily member 13C), and LTβR (tumor necrosis factor receptor superfamily member 3) play a pivotal role in non-small-cell lung cancer (NSCLC). To further evaluate their role in NSCLC, *CD40* rs1883832 (T>C), *BAFFR* rs7290134 (A>G), and *LTβR* rs10849448 (A>G) single-nucleotide polymorphisms (SNPs) were investigated regarding their impact in risk and clinical outcome of NSCLC patients.

**Methods:**

The three selected SNPs were evaluated in 229 NSCLC patients and 299 healthy controls, while CD40, BAFFR, and LTβR protein expression was assessed by immunohistochemistry in 96 tumor specimens from NSCLC patients.

**Results:**

In total, *CD40* rs1883832 was associated with NSCLC risk, with the T allele, after adjusting for cofactors, being related to increased risk (p = 0.007; OR 1.701). Moreover, the CT genotype was associated with increased risk (p = 0.024; OR 1.606) and poorer 5-year overall survival (OS) after adjusting for cofactors (p = 0.001, HR 1.829), while CC was associated with higher CD40 expression in tumorous cells (p = 0.040) and in stromal cells (p = 0.036). In addition, AA homozygotes for the *LTβR* rs10849448 had increased risk for NSCLC in multivariate analysis (p = 0.008; OR, 2.106) and higher LTβR membranous expression (p = 0.035). Although *BAFFR* rs7290134 was associated with BAFFR membranous expression (p = 0.039), *BAFFR* rs7290134 was not associated with neither the disease risk nor the prognosis of NSCLC patients.

**Conclusions:**

In conclusion, *CD40* rs1883832 and *LTβR* rs10849448 seem to be associated with increased risk for NSCLC, while *CD40* rs1883832 is also associated with OS of patients with NSCLC.

## Introduction

The last decade, NF-κB (nuclear factor kappa-light-chain-enhancer of activated B cells) has attracted interest regarding its role in NSCLC (non-small-cell lung cancer) (1). It has been documented that the main effectors of the classical, NF-κB1 and RelA (transcription factor P65), as well as of the alternative pathway, NF-κB2 and RelB (transcription factor RelB), are overexpressed and have prognostic value in NSCLC (1–5). On the contrary, published data on the pathobiology and the clinical significance in NSCLC of the surface receptors CD40 (tumor necrosis factor receptor superfamily member 5), BAFFR (tumor necrosis factor receptor superfamily member 13C), and LTβR (tumor necrosis factor receptor superfamily member 3), which mainly leads to signal transduction through the NF-κB alternative pathway, are limited. Recently, our group has reported that these receptors are expressed in NSCLC revealing significant clinical associations (6). According to our studies as well as to other studies, CD40 expression in lung cancer patients has been associated with metastatic progression (7) as well as with prognosis (6, 8). Similarly, BAFFR expression in NSCLC has also been reported to be deregulated (6), while its expression in CAFs (cancer-associated fibroblasts) has interestingly been associated with overall survival (OS) and response to platinum-based chemotherapy in NSCLC (9).

More limited are also the published data on genetic variations of *CD40*, *BAFFR*, and *LTβR* and especially of the three single-nucleotide polymorphisms (SNPs) *CD40* rs1883832 (T>C), *BAFFR* rs7290134 (A>G), and *LTβR* rs10849448 (A>G), which were evaluated in the current study, as well as their potent clinical value in NSCLC. In particular, *CD40* SNP rs1883832 has been associated with many nonmalignant clinical entities, such as atherosclerosis (10), acute coronary syndrome (11, 12), ischemic stroke (13), chronic obstructive pulmonary disease (14), chronic HBV infection (15), and later onset of Graves’ disease (16). It has also been related to cervical carcinoma in a subset of a Malaysian population (17) and to sporadic breast cancer risk in Chinese Han women (18). Interestingly, in a small study in the Chinese population, it has also been correlated with the susceptibility to lung cancer (19). Regarding the *BAFFR* rs7290134, no association with the risk for CLL (chronic lymphocytic leukemia) was found (20, 21), while no study has been published regarding its role in epithelial tumors. In addition, the published data regarding the clinical value of *LTβR* rs10849448 (A>G) refer to its association with individuals’ risk of undergoing tonsillectomy (22) and juvenile idiopathic arthritis (23), while no data exist regarding its significance in cancer.

In the present study, we present our findings on the clinical significance of *CD40* rs1883832 (T>C), *BAFFR* rs7290134 (A>G), and *LTβR* rs10849448 (A>G) SNPs in NSCLC patients in regard to the risk over NSCLC initiation and OS.

## Materials and Methods

### Study Design, Population, Tissue Specimens, and Data Collection

The current study was performed following Helsinki Declaration on ethical principles for medical research (2013) (24). In this study, we investigated the selected SNPs of *CD40*, *BAFFR*, and *LTβR* in relation to clinicopathological data, protein expression of the same molecules, and the clinical outcome of NSCLC patients. Initially, the selected SNPs were studied in a retrospectively collected group which was followed by a second prospectively collected patient cohort. Although our preliminary results from the retrospective group were validated in the prospective group, statistical analysis presented here was performed in the pool of the cases to achieve more robust statistical results.

Data (clinicopathological, disease outcome, and vital status) used in the analysis were obtained from pathology reports, from medical records, or through direct communication with the patients. Survival outcome was evaluated after the 60-month follow-up period. Clinicopathological characteristics of our cohort and relevant information are included in **Table 1**.

**Table 1:** Clinicopathological characteristics of patients of this study.

Clinicopathological characteristics	*Retrospectively collected cases (Group R)*	*Prospectively collected cases (Group P)*	*Total cases (Group PR)*
	*Cases*	*Cases*	*Cases*
	*n* (%)	*n* (%)	*n* (%)
**Total**	109 (100)	120 (100)	229 (100)
**Age (years)** Median (range)	67 (46–84)	66.5 (41–84)	67 (41–84)
Gender			
Total	109 (100)	120 (100)	229 (100)
Male	100 (91.7)	108 (90.0)	208 (90.8)
Female	9 (9.3)	12 (10.0)	21 (9.2)
Smoking (pack-years)			
Total	109 (100)	120 (100)	229 (100)
Cases (%)	47 (43.1)	43 (35.8)	90 (39.3)
Mean (range)	89.4 (20–165)	84.65 (10–200)	87.1 (10–200)
NA	62 (56.9)	77 (64.2)	139 (60.7)
Primary location			
Total	109 (100)	120 (100)	229 (100)
Left lung	49 (45.0)	51 (42.5)	100 (43.7)
Right lung	60 (55.0)	61 (50.8)	121 (52.8)
NA	–	8 (6.7)	8 (3.5)
Histology			
Total	109 (100)	120 (100)	229 (100)
Squamous	65 (59.6)	46 (38.3)	111 (48.5)
Adenocarcinoma	36 (33.0)	62 (51.7)	98 (42.8)
Large carcinoma	7 (6.4)	5 (4.2)	12 (5.2)
NA	1 (0.9)	7 (5.8)	8 (0.04)
Stage			
Total	109 (100)	120 (100)	229 (100)
I	37 (33.9)	21 (17.5)	58 (25.3)
II	32 (29.4)	19 (15.8)	51 (22.3)
III	37 (33.9)	32 (26.7)	69 (30.1)
IV	2 (1.8)	37 (30.8)	39 (17.0)
NA	1 (0.9)	11 (9.2)	12 (5.2)
Grade			
Total	109 (100)	120 (100)	229 (100)
I	4 (3.7)	2 (1.7)	6 (2.6)
II	45 (41.3)	40 (33.3)	85 (37.1)
III	52 (47.7)	38 (31.7)	90 (39.3)
NA	8 (7.3)	40 (33.3)	48 (21)
Maximum diameter (cm)			
Total	109 (100)	120 (100)	229 (100)
Cases (%)	106 (97.2)	81 (67.5)	187 (81.7)
Mean (range)	5.27 (0.70–21.0)	4.33 (1.0–10.0)	4.87 (0.70–21.0)
NA	3 (2.8)	39 (32.5)	42 (18.3)
Lymph node infiltration			
Total	109 (100)	120 (100)	229 (100)
No	56 (51.4)	35 (29.2)	91 (39.7)
Yes	49 (45.0)	31 (25.8)	80 (34.9)
NA	4 (3.7)	54 (45.0)	58 (25.3)
Metastasis (adrenals)			
Total	109 (100)	120 (100)	229 (100)
No	21 (19.3)	15 (12.5)	36 (15.7)
Yes	4 (4.7)	12 (10)	16 (7.0)
NA	84 (77.1)	93 (77.5)	177 (77.3)
Metastasis (liver)			
Total	109 (100)	120 (100)	229 (100)
No	22 (20.2)	12 (10.0)	34 (14.8)
Yes	3 (2.8)	17 (14.2)	20 (8.7)
NA	84 (77.1)	91 (75.8)	175 (76.4)
Metastasis (brain)			
Total	109 (100)	120 (100)	229 (100)
No	22 (20.2)	12 (10.0)	34 (14.8)
Yes	7 (6.4)	27 (22.5)	34 (14.8)
NA	80 (73.4)	81 (67.5)	161 (70.3)
Metastasis (bone)			
Total	109 (100)	120 (100)	229 (100)
No	16 (14.7)	14 (11.7)	30 (13.1)
Yes	13 (11.9)	26 (21.7)	39 (17.0)
NA	80 (73.4)	80 (66.7)	160 (69.9)
Metastasis (adrenals–liver–brain–bones)			
Total	109 (100)	120 (100)	229 (100)
No	7 (6.4)	6 (5.0)	13 (5.7)
Yes	25 (22.9)	49 (40.8)	74 (32.3)
NA	77 (70.6)	65 (54.2)	142 (62.0)
Survival (2 years)			
Total	109 (100)	120 (100)	229 (100)
Dead	42 (38.5)	63 (52.5)	105 (45.9)
Alive	65 (59.6)	52 (43.3)	117 (51.1)
NA	2 (1.8)	5 (4.2)	7 (3.1)
Survival (3 years)			
Total	109 (100)	120 (100)	229 (100)
Dead	58 (53.2)	77 (64.2)	135 (59.0)
Alive	47 (43.1)	37 (30.8)	84 (36.7)
NA	4 (3.7)	6 (5.0)	10 (4.4)
Survival (5 years)			
Total	109 (100)	120 (100)	229 (100)
Dead	67 (61.5)	90 (75.0)	157 (68.6)
Alive	38 (34.9)	24 (20.0)	62 (27.1)
NA	4 (3.7)	6 (5.0)	10 (4.4)
Relapse			
Total	109 (100)	120 (100)	229 (100)
No	9 (8.3)	1 (0.8)	10 (4.4)
Yes	17 (15.6)	12 (10.0)	29 (12.7)
NA	83 (76.1)	107 (89.2)	190 (83.0)

NA, not available or no further specifically categorized (e.g., NSCLC vs. squamous histology).

Histopathologically or cytologically confirmed NSCLC (squamous cell carcinoma or adenocarcinoma or large-cell carcinoma) patients were exclusively enrolled in the study. Totally, 229 previously untreated patients with confirmed NSCLC of all ages were enrolled, with 109 of them being retrospectively collected (Group R). Tissue specimens from the patients were collected from the archives of the Department of Pathology of the University Hospital of Patras, Greece: tumor-adjacent, nonmalignant, and macroscopically normal tissue specimens. Additionally, peripheral blood specimens from 120 newly diagnosed NSCLC patients, which were managed from 2008 to 2010, were prospectively collected (Group P). All cases were diagnosed and medically managed at the Division of Medical Oncology and the Department of Cardiothoracic Surgery at the University Hospital of Patras between 2004 and 2015. All patients of the study were treated based on the standard-of-care treatment options according to disease characteristics, the comorbidities, and the performance status following the current treatment guidelines. Uncertain histology, non-Greek Caucasian ethnic origin, and past medical history with cancer were the exclusion criteria for this study.

A healthy control group was also used, including 299 peripheral blood specimens from healthy control donors (Group H) who were collected in the context of annual medical checkup. Groups of controls and patients were age- and sex-matched (**Table 2**). In this study, only individuals with Greek Caucasian origin were recruited in order to ensure the similarity of the genetic background. Past medical history related to cancer or family history linked to any disease (including cancer) was set as exclusion criterion.

**Table 2:** Demographic characteristics of the healthy controls’ and NSCLC patients’ groups.

Groups (N)	Patients group (229)	Controls group (299)	p value
Age (years)	67 (41–84)	64 (30–95)	0.001
Gender (N males/females)	208/21	157/142	<0.001

### Selection of SNPs

The selection of the particular SNPs located within the *CD40*, *BAFFR*, and *LTβR* genes was performed by reviewing literature (PubMed, Google Scholar) using as keywords the words SNPs, *CD40*, *BAFFR*, *LTβR*, cancer, NSCLC, and lung cancer. Five percent was set as the cutoff point for minor allele frequencies, and the coefficient of determination r^2^ = 0.8 was captured. Genomic location, minor allele frequencies (MAF), and other specific characteristics of SNPs are presented in **Table 3**.

**Table 3:** Studied SNP information.

Gene	Gene location	Rs number	Base change	Genomic position (forward strand)	Genotyping success rate	Minor allele frequency		
						Control	1000genomes-CEU	1000genomes TSI-
CD40	5 prime UTR	rs1883832	T/C	20:46118343	99.8	0.441 (T)	0.232 (T)	0.299 (T)
BAFFR	3 prime UTR	rs7290134	A/G	22:41925247	100	0.187 (G)	0.247 (G)	0.215 (G)
LtBR	5 prime UTR	rs10849448	A/G	12:6384185	100	0.313 (A)	0.227 (A)	0.294 (A)

### DNA Isolation

The commercial kit “QIAamp Blood Mini Kit” (Qiagen Ltd., Crawley, UK) was used for the extraction of genomic DNA from whole blood samples from 124 cancer patients and 279 healthy donors according to the manufacturer’s instructions. Furthermore, DNA was also isolated from 148 tumor adjacent, nonmalignant FFPE, tissue specimens using the “QIAamp DNA FFPE Tissue Kit” (Qiagen Ltd., Crawley, UK). DNA was stored at -20°C until required.

### Genotyping

Genotyping was performed using real-time PCR followed by high-resolution melt curve (HRM) analysis on a StepOnePlus™ real-time PCR system (Thermo Fisher Scientific, Waltham, MA USA). Reactions were performed using SNP-specific primers (*CD40* rs1883832: forward 5′-GCCTGGTCTCACCTCGC-3′ and reverse 5′-GCCCCAGAGGACGCAC-3′; *BAFFR* rs7290134: forward 5′-GCTGAATGCTGTGGTCTGTAGTG-3′ and reverse 5′-CATGCACATGCCCTCTTTCTG-3′; *LtBR* rs10849448 forward 5′-CGGCCAGCTCGCTCCAC-3′ and reverse 5′-GCCTCCAGGGCTCCCA-3′) and MeltDoctor™ HRM Master Mix (Thermo Fisher Scientific, Waltham, MA USA). HRM genotyping data were analyzed using the High-Resolution Melt Software v3.0 (Applied Biosystems, Thermo Fisher Scientific, Waltham, MA USA).

### Sequencing

Further validation of the genotyping method as well as genotyping in some subgroups of participants due to the observed deviation from the Hardy–Weinberg equilibrium (HWE) was achieved through sequencing of several samples, representative of all three genotypes. The sequences of the primers and PCR conditions that were used for sequencing can be provided upon request. Sequencing was performed at Cemia SA (University of Thessaly, Greece). All obtained sequences were in agreement with the genotyping results of our method.

### Immunohistochemical Analysis

CD40, BAFFR, and LTβR protein expression was studied by immunohistochemistry in a cohort of patients, the vast majority of whom had undergone curative resection of a lung tumor in the University Hospital of Patras between 2005 and 2010 (**Table 4**). Selection of patients was serially and retrospectively done while patient data analysis was performed blindly based on the archive and database of the Pathology Department of the University Hospital of Patras. Invasive, formalin-fixed paraffin-embedded (FFPE) NSCLC tissue specimens as well as adjacent non-neoplastic lung parenchyma were retrieved.

**Table 4:** Clinicopathological characteristics and survival data of NSCLC patients studied by immunohistochemistry.

Clinicopathological characteristics	*Total cases n* (%)
Total	96 (100)
Age (years) Median (range)	67 (44–84)
Gender	
Total	96 (100)
Male	90 (93.8)
Female	6 (6.2)
Smoking (pack-years)	
Total	96 (100)
Cases (%)	39 (40.6)
Mean (range)	90.92 (20–165)
NA	57 (59.4)
Primary location	
Total	96 (100)
Left lung	38 (39.6)
Right lung	58 (60.4)
NA	–
Histology	
Total	96 (100)
Squamous	56 (58.3)
Adenocarcinoma	32 (33.3)
Large carcinoma	8 (8.3)
NA	–
Stage	
Total	96 (100)
I	33 (34.4)
II	31 (32.3)
III	30 (31.3)
IV	2 (2.1)
NA	–
Grade	
Total	96 (100)
I	4 (4.2)
II	42 (43.8)
III	42 (43.8)
NA	8 (8.3)
Maximum diameter (cm)	
Total	96 (100)
Cases (%)	94 (98.0)
Mean (range)	5.34 (1.10–21.00)
NA	2 (2.0)
Lymph node infiltration	
Total	96 (100)
No	49 (51.0)
Yes	43 (45.8)
NA	3 (3.1)
Metastasis (adrenals)	
Total	96 (100)
No	17 (17.7)
Yes	1 (1.0)
NA	78 (81.3)
Metastasis (liver)	
Total	96 (100)
No	17 (17.7)
Yes	3 (3.1)
NA	76 (79.2)
Metastasis (brain)	
Total	96 (100)
No	17 (17.7)
Yes	6 (6.3)
NA	73 (76.0)
Metastasis (bones)	
Total	96 (100)
No	13 (13.5)
Yes	11 (11.5)
NA	72 (75.0)
Metastasis (adrenals–liver–brain–bones)	
Total	96 (100)
No	6 (6.3)
Yes	21 (21.9)
NA	69 (71.9)
Survival (2 years)	
Total	96 (100)
Dead	37 (38.5)
Alive	57 (59.4)
NA	2 (2.1)
Survival (3 years)	
Total	96 (100)
Dead	50 (52.1)
Alive	42 (43.8)
NA	4 (4.2)
Survival (5 years)	
Total	96 (100)
Dead	59 (61.5)
Alive	33 (34.4)
NA	4 (4.2)
Relapse	
Total	96 (100)
No	8 (8.3)
Yes	14 (14.6)
NA	74 (77.1)

NA, data not available or unknown.

Mouse monoclonal antibodies against CD40 and BAFFR and a rabbit polyclonal antibody against LTβR were used for antigen detection. Specific conditions regarding clonality, clone, dilution, antigen retrieval, and incubation time have been described in depth in previous publications (6); thus, here they are presented briefly (**Table 5**). Detection and visualization were performed using the EnVision Detection Kit (DAKO) and diaminobenzidine (DAB) chromogen following the manufacturer’s instructions. In addition, dehydrated Harris’ hematoxylin was used to counterstain the sections.

**Table 5:** Primary antibodies and their clonality, clone, dilution, antigen retrieval, and incubation time information.

Antibody	Clonality	Company	Catalogue number	Clone	Dilution	Antigen retrieval conditions	Incubation time
LTβR	P	Abcam	Ab193449		1:750	8 mM sodium citrate, pH 6.0	Overnight 4°C
CD40	M	SANTA CRUZ	Sc-13528	LOB-11	1:20	1.2 mM EDTA, pH 8.0	Overnight 4°C
BAFFR	M	SANTA CRUZ	Sc-32774	11C1	1:20	1.2 mM EDTA, pH 8.0	Overnight 4°C

### Evaluation of Immunohistochemistry

An evaluation of immunohistochemical staining has been described in detail in a previous publication (6). Briefly, an experienced pathologist (VT) assessed and scored each slide in a blind fashion. Tumor as well as stromal cells (myofibroblasts), tumor-infiltrating lymphocytes (TILs), and tumor-associated macrophages (TAMs) were scored. An initial selection of representative areas was performed at low (×100) magnification. A minimum number of 1,000 cells per tissue section was counted at a ×400 magnification. Cytoplasmic staining was evaluated for both epithelial and stromal cells, while membranous staining was performed only for epithelial cells. Microphotographs were taken by using a Nikon DXM1200C digital camera mounted on a Nikon Eclipse 80i microscope and ACT-1C software (Nikon Instruments Inc., Melville, NY, USA).

### Statistical Analysis

Statistical analysis was performed by using Statistical Package for Social Sciences version 17 (SPSS, Chicago, IL, USA). Categorical nominal variables were evaluated using the chi-square test or Fisher’s exact test. The T test was used for continuous variables with normal distribution. Analysis by using the Kruskal–Wallis or Mann–Whitney test was performed for ordinal or continuous data. Spearman’s correlations were used to assess associations between variables. The Kaplan–Meier method and the log-rank test were used for plotting of survival rates and their comparison, respectively. p<0.05 was considered statistically significant for all comparisons.

## Results

### Frequencies of Genotypes and Alleles Across Subpopulations

In the current study, genotyping of the three studied polymorphisms was achieved in the vast majority of patients and controls, who were enrolled in the study. In particular, genotyping for *CD40* rs1883832 (T>C) was successfully performed in 228 patients and all healthy controls (**Table 6**). The frequencies of the three *CD40* rs1883832 genotypes (TT, CT, CC) were 23.6%, 26.8%, and 49.6%, in the NSCLC cases and 24.4%, 39.5%, and 36.1% in healthy controls, respectively. In addition, *BAFFR* rs7290134 (A>G) was successfully detected in all patients and 298 healthy controls. The frequencies of the three *BAFFR* rs7290134 (A>G) genotypes (AA, AG, GG) were 64.2%, 31.9%, and 3.9% in lung cancer patients and 62.4%, 3.9%, and 5.7% in healthy controls, respectively (**Table 7**). Furthermore, 223 NSCLC patients and all healthy controls were genotyped for *LTβR* rs10849448 (A>G). The frequencies for the three genotypes AA, AG, and GG of *LTβR* rs10849448 (A>G) were 10.8%, 27.4%, and 61.8% in the NSCLC subcohort and 18.4%, 25.8%, and 55.8% in healthy controls, respectively (**Table 8**).

**Table 6:** Relationships between clinicopathological variables of NSCLC patients and *CD40* rs1883832 genotypes.

Clinicopathological characteristics	*Patients*	*Genotypes*		*p-value*	*Genotypes*		*p-value*
	*n* (%)	CC	CT+TT		TT	CC+CT	
Total	229 (100)						
Genotyped	228 (99.6)	113 (49.3)	115 (50.2)		54 (23.6)	174 (76.0)	
NA	1 (0.4)						
Age (years) Mean (range)	65 (40–84)						
Genotyped	228 (100)	39 (17.1)	74 (32.5)	0.584	22 (9.6)	61 (26.8)	0.518
<65	83 (36.4)	44 (19.3)	71 (31.3)		32 (14.0)	113 (49.6)	
>=65	145 (63.6)						
Gender							
Genotyped	228 (100)	101 (44.3)	106 (46.5)	0.500	50 (21.9)	157 (68.9)	0.789
Male	207 (90.8)	12 (5.3)	9 (3.9)		4 (1.8)	17 (7.5)	
Female	21 (9.2)						
Smoking (pack-years)							
Genotyped	228 (100)	48 (21.1)	41 (18.0)	0.840	21 (9.2)	68 (29.8)	0.927
Cases	89 (39.0)	85.18 (10–165)	90.30 (15–200)		88.19 (15–200)	87.34 (10–180)	
Mean (range)	87.12 (10–200)	65 (28.5)	74 (32.5)		33 (14.5)	106 (46.5)	
να	139 (61.0)						
Primary location							
Genotyped	228 (100)	46	54	0.343	23	77	1.000
Left lung	100 (43.9)	64	56		28	92	
Right lung	120 (52.6)	3 (1.3)	5 (2.2)		3 (1.3)	5 (2.2)	
NA	8 (3.5)						
Histology							0.958
Genotyped	228 (100)	51	60	0.572	27	84	
Squamous	111	52	46		23	75	
Adenocarcinoma	98	5	6		3	8	
Large carcinoma	11	5 (2.2)	3 (1.3)		1 (0.4)	7 (3.1)	
NA	8 (3.5)						
Stage							
Genotyped	228 (100)	27	31	0.226	13	45	0.754
I	58	30	20		10	40	
II	50	35	34		19	50	
III	69	15	24		8	31	
IV	39	6 (2.6)	6 (2.6)		4 (1.8)	8 (3.5)	
NA	12 (5.3)						
Grade							
Genotyped	228 (100)	4	2	0.570	0	6	0.262
I	6	41	44		23	62	
II	85	48	41		19	70	
III	89	20 (8.8)	28 (12.3)		12 (5.3)	36 (15.8)	
NA	48 (21.1)						
Maximum diameter (cm)							
Genotyped	228 (100)	95	91	0.435	44	142	0.618
Cases (%)	186	5.02 (1.20–14)	4.68 (0.7–21)		4.8 (0.7–21)	4.88 (1.1–14)	
Mean (range)	4.87 (0.7–21)	18 (7.9)	24 (10.5)		10 (4.4)	32 (14.0)	
NA	42 (18.4)						
Lymph node infiltration							
Genotyped	228 (100)	43	48	0.286	25	66	0.287
No	91	44	35		16	63	
Yes	79	26 (11.4)	32 (14.0)		13 (5.7)	45 (19.7)	
NA	58 (25.4)						
Metastasis (adrenals)^a^							
Genotyped	228 (100)	18	17	0.384	8	27	0.730
No	35	6	10		5	11	
Yes	16	89 (39.0)	88 (38.5)		41 (18.0)	136 (59.6)	
NA	177 (77.6)						
Metastasis (liver)^a^							
Genotyped	228 (100)						
No	30	16	17	1.000	8	25	1.000
Yes	20	9	11		5	15	
NA	175 (76.8)	8 (38.6)	87 (38.2)		4 (18.0)	134 (58.8)	
Metastasis (brain)^a^							
Genotyped	228 (100)	19	15	0.144	8	26	0.784
No	34	12	21		9	24	
Yes	33	82 (36.0)	79 (34.6)		37 (16.2)	124 (54.4)	
NA	161 (70.6)						
**Metastasis (bones)** **^a^**							
Genotyped	228 (100)	16	13	0.087	7	22	0.786
No	29	13	26		11	28	
Yes	39	84 (36.8)	76 (33.3)		36 (15.8)	124 (54.4)	
NA	160 (70.2)						
Metastasis (adrenals–liver–brain–bones)^a^							
Genotyped	228 (100)	10	3	0.032	1	12	0.283
No	13	30	43		19	54	
Yes	73						
NA							
Survival (2 years)							
Genotyped	228 (100)	50	55	0.687	19	86	0.081
Dead	105	59	57		33	83	
Alive	116	4 (1.8)	3 (1.3)		2 (0.9)	5 (2.2)	
NA	7 (3.1)						
Survival (3 years)							
Genotyped	228 (100)	60	74	0.166	25	109	0.033
Dead	134	46	38		27	57	
Alive	84	7 (3.1)	3 (1.3)		2 (0.9)	8 (3.5)	
NA	10 (4.4)						
Survival (5 years)							
Genotyped	228 (100)	72	84	0.294	33	123	0.160
Dead	156	34	28		19	43	
Alive	62	7 (3.1)	3 (1.3)		2 (0.9)	8 (3.5)	
NA	10 (4.4)						

NA, data not available or unknown.

a Metastasis detected at follow-up, not at the time of sample collection.

**Table 7:** Relationships between clinicopathological variables of NSCLC patients and *BAFFR* rs7290134 genotypes.

Clinicopathological characteristics	*Patients*	*Genotypes*		*p-value*	*Genotypes*		*p-value*
	*n* (%)	AA	AG+GG		GG	AG+AA	
Total	229 (100)						
Genotyped	229 (100)	147 (64.2)	82 (35.8)		9 (3.9)	220 (96.1)	
NA	0 (0)						
Age (years) Mean (range)							
Genotyped	229 (100)	49 (21.4)	35 (15.3)	0.198	4 (1.7)	80 (34.9)	0.728
<65	84 (36.7)	98 (42.8)	47 (20.5)		5 (2.2)	140 (61.1)	
>=65	145 (63.3)						
Gender							
Genotyped	229 (100)	133 (38.1)	75 (32.8)	1.000	6 (2.6)	202 (88.2)	0.039
Male	208 (90.8)	14 (6.1)	7 (3.1)		3 (1.3)	18 (7.9)	
Female	21 (9.2)						
Smoking (pack-years)							
Genotyped	229 (100)	57 (24.9)	33 (14.4)	0.923	6 (2.6)	84 (36.7)	0.752
Cases	90 (39.3)	86.70 (10–180)	87.85 (25–200)		85.00 (30–153)	87.27 (10–200)	
Mean (range)	87.12 (10–200)	90 (39.3)	49 (21.4)		3 (1.3)	136 (59.4)	
NA	139 (60.7)						
Primary location							
Genotyped	229 (100)	62 (27.1)	38 (16.6)	0.779	5 (2.2)	95 (41.5)	0.735
Left lung	100 (43.7)	78 (34.1)	43 (18.8)		4 (1.7)	117 (51.1)	
Right lung	121 (52.8)	7 (3.1)	1 (0.4)		0 (0)	8 (3.5)	
NA	8 (3.5)						
Histology							
Genotyped	229 (100)	72 (31.4)	39 (17.0)	0.353	4 (1.7)	107 (46.7)	0.772
Squamous	111 (48.5)	61 (26.6)	37 (16.2)		4 (1.7)	94 (41.0)	
Adenocarcinoma	98 (42.8)	10 (4.4)	2 (0.9)		0 (0)	12 (5.2)	
Large carcinoma	12 (5.2)	4 (1.7)	4 (1.7)		1 (0.4)	7 (3.1)	
NA	8 (3.5)						
Stage							
Genotyped	229 (100)	34 (14.8)	24 (10.5)	0.469	2 (0.9)	56 (24.5)	0.664
I	58 (25.3)	30 (13.1)	21 (9.2)		2 (0.9)	49 (21.4)	
II	51 (22.3)	46 (20.1)	23 (10.0)		2 (0.9)	67 (29.3)	
III	69 (30.1)	28 (12.2)	11 (4.8)		3 (1.3)	36 (15.7)	
IV	39 (17.0)	9 (3.9)	3 (1.3)		3 (1.3)	9 (3.9)	
NA	12 (5.2)						
Grade							
Genotyped	229 (100)	5 (2.2)	1 (4.4)	0.377	1 (0.4)	5 (2.2)	0.074
I	6 (2.6)	50 (21.8)	35 (15.3)		4 (1.7)	81 (35.4)	
II	85 (37.1)	59 (25.8)	31 (13.5)		1 (4.4)	89 (38.9)	
III	90 (39.3)	33 (14.4)	15 (6.6)		3 (1.3)	45 (19.7)	
NA	48 (21.0)						
Maximum diameter (cm)							
Genotyped	229 (100)	116 (50.1)	71 (31.0)	0.037	6 (2.6)	181 (79.0)	0.371
Cases (%)	187 (81.7)	5.25 (0.7–21)	4.24 (1.1–9.5)		5.3 (3–7.5)	4.85 (0.7–21)	
Mean (range)	4.87 (0.7–21)	31 (13.5)	11 (4.8)		3 (1.3)	39 (17.0)	
NA	42 (18.3)						
Lymph node infiltration							
Genotyped	229 (100)	56 (24.5)	35 (15.2)	0.874	4 (1.7)	87 (38.0)	0.373
No	91 (39.7)	51 (22.3)	29 (12.7)		1 (0.4)	79 (34.5)	
Yes	80 (34.9)	40 (17.5)	18 (7.9)		4 (1.7)	54 (23.6)	
NA	58 (25.3)						
Metastasis (adrenals)^a^							
Genotyped	229 (100)	26 (11.4)	10 (4.4)	0.301	1 (0.4)	35 (15.3)	1.000
No	36 (15.7)	14 (6.1)	2 (0.9)		0 (0)	16 (7.0)	
Yes	16 (7.0)	107 (46.7)	70 (30.6)		8 (3.5)	169 (73.8)	
NA	177 (77.3)						
Metastasis (liver)^a^							
Genotyped	229 (100)						1.000
No	34 (14.8)	24 (10.5)	10 (4.4)	0.329	1 (0.4)	33 (14.4)	
Yes	20 (8.7)	17 (7.4)	3 (1.3)		1 (0.4)	19 (8.3)	
NA	175 (76.4)	106 (46.3)	69 (30.1)		7 (3.1)	168 (73.4)	
Metastasis (brain)^a^							
Genotyped	229 (100)	25 (10.9)	9 (3.9)	0.791	0 (0)	34 (14.8)	0.114
No	34 (14.8)	23 (10.0)	11 (4.8)		4 (1.7)	30 (13.1)	
Yes	34 14.8)	99 (43.2)	62 (27.1)		5 (2.2)	156 (68.1)	
NA	161 (70.3)						
Metastasis (bones)^a^							
Genotyped	229 (100)	21 (9.2)	9 (3.9)	0.797	1 (0.4)	29 (12.7)	1.000
No	30 (13.1)	25 (10.9)	14 (6.1)		2 (0.9)	37 (16.2)	
Yes	39 (17.0)	101 (44.1)	59 (25.8)		6 (2.6)	154 (67.2)	
NA	160 (69.9)						
Metastasis (adrenals–liver–brain–bones)^a^							
Genotyped	229 (100)	9 (3.9)	4 (1.7)	1.000	0 (0)	13 (5.7)	1.000
No	13 (5.7)	50 (21.8)	24 (10.7)		4 (1.7)	70 (30.6)	
Yes	74 (32.3)	88 (39.3)	54 (23.6)		5 (2.2)	137 (59.8)	
NA	142 (62.0)						
Survival (2 years)							
Genotyped	229 (100)	74 (32.3)	31 (13.5)	0.092	4 (1.7)	101 (44.1)	1.000
Dead	105 (45.9)	69 (30.1)	48 (21.0)		4 (1.7)	113 (49.3)	
Alive	117 (51.1)	4 (1.7)	3 (1.3)		1 (0.4)	6 (2.6)	
NA	7 (3.1)						
Survival (3 years)							
Genotyped	229 (100)	91 (39.7)	44 (19.2)	0.249	6 (2.6)	129 (56.3)	0.714
Dead	135 (59.0)	50 (21.8)	34 (14.8)		2 (0.9)	82 (35.8)	
Alive	84 (36.7)	6 (2.6)	4 (1.7)		1 (0.4)	9 (3.9)	
NA	10 (4.4)						
Survival (5 years)							
Genotyped	229 (100)	107 (46.7)	50 (21.8)	0.084	6 (2.6)	151 (66.0)	1.000
Dead	157 (68.6)	34 (14.8)	28 (12.2)		2 (0.9)	60 (26.2)	
Alive	62 (27.1)	6 (2.6)	4 (1.7)		1 (0.4)	9 (3.9)	
NA	10 (4.4)						

NA, data not available or unknown.

a Metastasis detected at follow-up, not at the time of sample collection.

**Table 8:** Relationships between clinicopathological variables of NSCLC patients and *LtBR* rs10849448 genotypes.

Clinicopathological characteristics	*Patients*	*Genotypes*		*p-value*	*Genotypes*		*p-value*
	*n* (%)	GG	AG+AA		AA	AG+GG	
Total	229 (100)						
Genotyped	223 (97.4)	138 (61.9)	85 (38.1)		24 (10.8)	199 (89.2)	
NA	6 (2.6)						
Age (years) Mean (range)	65 (40–84)						
Genotyped	223 (100)	44 (19.7)	37 (16.6)	0.087	9 (4.0)	72 (32.3)	1.000
<65	81 (36.3)	94 (42.2)	48 (21.5)		15 (6.7)	127 (57.0)	
>=65	142 (63.7)						
Gender							
Genotyped	223 (100)	126 (56.5)	77 (34.5)	1.000	22 (9.9)	181 (81.2)	1.000
Male	203 (91.0)	12 (5.4)	8 (3.6)		2 (0.9)	18 (8.1)	
Female	20 (9.0)						
Smoking (pack–years)							
Genotyped	223 (100)	51 (22.7)	35 (15.7)	0.748	11 (4.9)	75 (33.6)	0.564
Cases	86 (38.6)	87.22 (10–180)	91.44 (15–200)		98.64 (30–200)	87.51 (10–180)	
Mean (range)	87.12 (10–200)	87 (39)	50 (22.4)		13 (5.8)	124 (55.6)	
NA	137 (61.4)						
Primary location							
Genotyped	223 (100)	59 (26.5)	38 (17.0)	0.889	10 (4.5)	87 (39.0)	1.000
Left lung	97 (43.5)	73 (32.7)	45 (20.2)		13 (5.8)	105 (47.1)	
Right lung	118 (52.9)	6 (2.7)	2 (0.9)		1 (0.4)	7 (3.1)	
NA	8 (3.6)						
Histology							
Genotyped	223 (100)	73 (32.7)	35 (15.7)	0.202	6 (2.7)	102 (45.7)	0.065
Squamous	108 (48.4)	55 (24.7)	42 (18.8)		15 (6.7)	82 (36.8)	
Adenocarcinoma	97 (43.5)	5 (2.2)	5 (2.2)		1 (0.4)	9 (4.0)	
Large carcinoma	10 (4.5)	5 (2.2)	3 (1.3)		2 (0.9)	6 (2.7)	
NA	8 (3.6)						
Stage							
Genotyped	223 (100)	31 (13.9)	24 (10.8)	0.631	6 (2.7)	49 (22.0)	0.216
I	55 (24.7)	28 (12.6)	21 (9.4)		7 (3.1)	42 (18.8)	
II	49 (22.0)	45 (20.2)	23 (10.3)		3 (1.3)	65 (29.1)	
III	68 (30.5)	25 (11.2)	14 (6.3)		6 (2.7)	33 (14.8)	
IV	39 17.5)	9 (4.0)	3 (1.3)		2 (0.9)	10 (4.5)	
NA	12 (5.4)						
Grade							
Genotyped	223 (100)	4 (1.8)	2 (0.9)	0.371	2 (0.9)	4 (1.8)	0.048
I	6 (2.7)	45 (20.2)	36 (16.1)		10 (4.5)	71 (31.8)	
II	81 (36.3)	58 (26.0)	30 (13.5)		5 (2.2)	83 (37.2)	
III	88 (39.5)	31 (13.9)	17 (7.6)		7 (3.1)	41 (18.4)	
NA	48 (21.5)						
Maximum diameter (cm)							
Genotyped	223 (100)	111 (49.8)	70 (31.4)	0.435	18 (8.1)	163 (73.1)	0.618
Cases (%)	187	4.89 (1–14)	4.78 (0.7–21)		4 (0.7–10)	4.94 (1–21)	
Mean (range)	4.87 (0.7–21)	27 (12.1)	15 (6.7)		6 (2.7)	36 (16.1)	
NA	36 (16.1)						
Lymph node infiltration							
Genotyped	223 (100)	51 (22.7)	35 (15.7)	0.752	9 (4.0)	77 (34.5)	1.000
No	86 (38.6)	49 (22.0)	30 (13.5)		8 (3.6)	71 (31.8)	
Yes	79 (35.4)	38 (17.0)	20 (9.0)		7 (3.1)	51 (22.9)	
NA	58 (26.0)						
Metastasis (adrenals)^a^							
Genotyped	223 (100)	18 (8.1)	17 (7.6)	0.015	5 (2.2)	30 (13.5)	1.000
No	35 (15.7)	14 (6.3)	2 (0.9)		2 (0.9)	14 (6.3)	
Yes	16 (7.2)	116 (52.0)	66 (29.6)		17 (7.6)	155 (69.5)	
NA	172 (77.1)						
Metastasis (liver)^a^							
Genotyped	223 (100)						
No	33 (14.8)	19 (8.5)	14 (6.3)	0.773	4 (1.8)	29 (13.0)	1.000
Yes	20 (9.0)	13 (5.8)	7 (3.1)		2 (0.9)	18 (8.1)	
NA	168 (75.3)	106 (47.5)	64 (28.7)		8 (3.6)	152 (68.2)	
Metastasis (brain)^a^							
Genotyped	223 (100)	19 (8.5)	15 (6.7)	1.000	5 (2.2)	29 (13.0)	0.752
No	34 (15.2)	18 (8.1)	15 (6.7)		6 (2.7)	27 (12.2)	
Yes	33 (14.8)	101 (45.3)	55 (24.7)		13 (5.8)	141 (63.2)	
NA	156 (70.0)						
Metastasis (bones)^a^							
Genotyped	223 (100)	17 (7.6)	12 (5.4)	0.802	3 (1.3)	26 (11.7)	0.721
No	29 (13.0)	24 (10.8)	14 (6.3)		6 (2.7)	32 (14.3)	
Yes	38 (17.0)	97 (43.5)	59 (26.5)		15 (6.7)	141 (63.2)	
NA	156 (70.0)						
Metastasis (adrenals–liver–brain–bones)^a^							
Genotyped	223 (100)	5 (2.2)	8 (3.6)	0.122	2 (0.9)	11 (4.9)	0.670
No	13 (5.8)	47 (21.1)	26 (11.7)		9 (4.0)	64 (28.7)	
Yes	73 (32.7)	86 (38.6)	51 (22.7)		13 (5.8)	124 (55.6)	
NA	137 (61.4)						
Survival (2 years)							
Genotyped	223 (100)	64 (28.7)	40 (17.9)	1.000	10 (4.5)	94 (42.2)	0.524
Dead	104 (46.6)	68 (30.5)	44 (19.7)		14 (6.3)	98 (43.9)	
Alive	112 (50.2)	6 (2.7)	1 (0.4)		0 (0)	7 (3.1)	
NA	7 (3.1)						
Survival (3 years)							
Genotyped	223 (100)	84 (37.7)	49 (22.0)	0.469	14 (6.3)	119 (53.4)	1.000
Dead	133 (59.6)	46 (20.6)	34 (15.2)		9 (4.0)	71 (31.8)	
Alive	80 (35.9)	8 (3.6)	2 (0.9)		1 (0.4)	9 (4.0)	
NA	10 (4.5)						
Survival (5 years)							
Genotyped	223 (100)	99 (44.4)	55 (24.7)	0.120	15 (6.7)	139 (62.3)	0.462
Dead	154 (69.1)	31 (13.9)	28 (12.6)		8 (3.6)	51 (2.2)	
Alive	59 (26.5)	8 (3.6)	2 (0.9)		1 (0.4)	9 (4.0)	
NA	10 (4.5)						

NA, data not available or unknown.

a Metastasis detected at follow-up, not at the time of sample collection.

### *CD40* rs1883832 (T>C) and LTβR rs10849448 (A>G) SNPs Were Associated With Increased NSCLC Risk

As shown in **Table 9**, a statistically significant difference was observed in the allele frequency of *CD40* rs1883832 (T) between lung cancer patients and controls (p=0.021) as well as between the frequencies of the studied genotypes (p=0.003). Statistically significant was also the difference between genotypes in NSCLC patients and healthy controls following the dominant model for the T allele (p = 0.002). In univariate analysis, T allele carriers (TT+CT) for *CD40* rs1883832 had a higher risk for lung cancer (p = 0.002; OR 1.738, 95% CI 1.224–2.468). The same relation remained statistically significant under a multivariate model, in which age, smoking history, and genotype for *CD40* rs1883832 SNP were entered as covariates (p = 0.007; OR 1.701, 95% CI 1.154–2.506). In addition, using an overdominant gene model (CT vs. CC+ TT), the CT genotype was also associated with increased risk for NSCLC not only in univariate analysis (p = 0.002; OR 1.785, 95% CI 1.228–2.594) but also after adjusting for cofactors (p = 0.024; OR 1.606, 95% CI 1.065–2.422).

**Table 9:** ORs and 95% CIs for NSCLC in relation to genotypes of studied SNPs.

Genotype	Cases n (%)	Controls n (%)	*p^a^*	^b^Crude OR (95% CI)	*p*	^c^Adjusted OR (95% CI)	*p*
Total	229	299	–	–	–	–	–
***CD40* rs1883832 (T>C)**	228	299	–	–	–	–	–
TT	54 (23.7)	73 (24.4)	**0.003**	1.414 (0.911–2.196)	0.122	1.508 (0.930–2.445)	0.096
CT	61 (26.8)	118 (39.5)		2.024 (1.348–3.039)	**0.001**	1.865 (1.192–2.919)	**0.006**
CC	113 (49.5)	108 (36.1)		1.000	**-**	1.000	–
TT+CT	115 (50.4)	191 (63.9)	**0.002**	1.738 (1.224–2.468)	**0.002**	1.701 (1.154–2.506)	**0.007**
vs. CC	113 (49.6)	108 (36.1)					
TT	54 (23.7)	73 (24.4)	0.846	1.041 (0.695–1.558)	0.846	1.156 (0.742–1.800)	0.523
vs. CT+CC	174 (76.3)	226 (75.6)					
CT	61 (26.8)	118 (39.5)	**0.002**	1.785 (1.228–2.594)	**0.002**	1.606 (1.065–2.422)	**0.024**
vs. TT+ CC	167 (73.2)	181 (60.5)					
T allele	169 (37.1)	264 (44.1)	**0.021**	1.342 (1.046–1.722)	**0.021**	1.384 (1.052–1.821)	**0.020**
C allele	287 (62.9)	334 (55.9)		0.745 (0.581–0.956)	**0.021**	0.723 (0.549–0.951)	**0.020**
***BAFFR* rs7290134 (A>G)**	229	298	–	–	–	–	–
AA	147 (64.2)	186 (62.4)	0.640	1.000	–	1.000	–
AG	73 (31.9)	95 (31.9)		1.029 (0.708–1.495)	0.883	1.054 (0.697–1.593)0	0.803
GG	9 (3.9)	17 (5.7)		1.493 (0.647–3.446)	0.348	1.647 (0.669–4.056)	0.278
AA vs	147 (64.2)	186 (62.4)	0.675	0.926 (0.648–1.325)	0.675	0.894 (0.603–1.326)	0.578
AG+GG	82 (35.8)	112 (37.6)					
AA+AG	220 (96.1)	281 (94.3)	0.351	0.676 (0.296–1.546)	0.354	0.666 (0.271–1.637)	0.376
vs. GG	9 (3.9)	17 (5.7)					
AG vs.	73 (31.9)	96 (32.1)	0.955	1.011 (0.699–1.462)	0.955	1.039 (0.691–1.561)	0.854
AA+GG	156 (68.1)	203 (67.9)					
A allele	367 (80.1)	467 (78.4)	0.482	0.898 (0.664–1.213)	0.482	0.864 (0.621–1.202)	0.385
G allele	91 (19.9)	129 (21.6)		1.114 (0.492–0.929)	0.482	1.158 (0.832–1.611)	0.385
***LTβR* rs10849448 (A>G)**	223	299	–	–	–	–	–
AA	24 (10.8)	55 (18.4)	0.054	1.894 (1.115–3.217)	**0.018**	2.051 (1.159–3.628)	**0.014**
AG	61 (27.4)	77 (25.8)		1.043 (0.696–1.563)	0.838	0.934 (0.593–1.473)	0.934
GG	138 (61.8)	167 (55.8)		1.000	–	1.000	–
AA vs	24 (10.8)	55 (18.4)	**0.016**	1.869 (1.117–3.127)	**0.017**	2.106 (1.210–3.667)	**0.008**
AG+GG	199 (89.2)	244 (81.6)					
AA+AG vs	85 (38.1)	132 (44.1)	0.167	0.779 (0.547–1.110)	0.167	1.255 (0.848–1.856)	0.256
GG	138 (61.9)	167 (55.9)					
AG	61 (27.4)	77 (25.8)	0.681	0.921 (0.622–1.364)	0.681	0.995 (0.978–1.013)	0.602
vs, GG+AA	162 (72.6)	222 (74.2)					
A allele	109 (24.4)	187 (31.3)	**0.015**	1.407 (1.067–1.855)	**0.016**	1.436 (1.060–1.945)	**0.019**
G allele	337 (75.6)	411 (68.7)		0.711 (0.539–0.937)	**0.016**	0.696 (0.514–0.943)	**0.019**

^a^p derives from the χ^2^ test and refers to the overall association of genotypes with NSCLC risk. ^b^p, OR, and 95% CI derived from logistic regression analysis using no cofactor. ^c^p, OR, and 95% CI derived from logistic regression analysis using age and gender as cofactors.

CI, confidence interval; OR, odds ratio.

Bold text indicates a statistically significant correlation with a p-value less than 0.05.

In addition, *LTβR* rs10849448 (A>G) SNP was also associated with the development of NSCLC. Particularly, homozygosity for the alternative allele A was related to higher risk for NSCLC in univariate (p=0.016; OR, 1.869; 95% 1.117–3.127) and multivariate analyses using age as cofactor (p=0.008; OR, 2.106; 95% 1.210–3.667). On the contrary, no association was observed between *BAFFR* rs7290134 SNP and the risk for NSCLC following any model.

### *CD40* rs1883832 (T>C) Was Associated With Overall Survival

**Figure 1:**
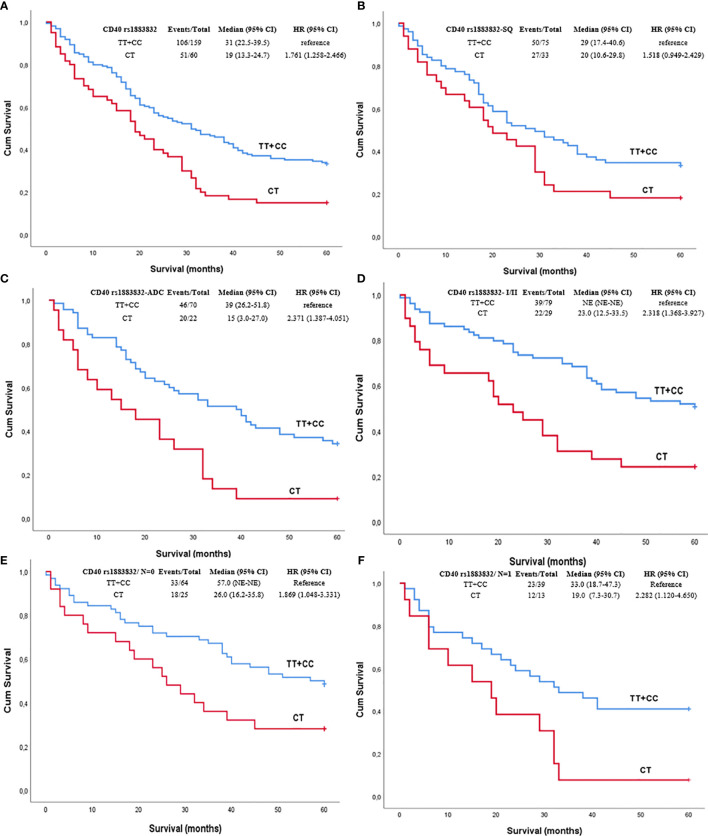
Kaplan–Meier curves depicting overall survival (OS) of NSCLC patients in relation to **(A)** *CD40* rs1883832 genotypes in the whole sub-cohort of NSCLC patients, **(B)** *CD40* rs1883832 genotypes in SQ patients, **(C)** *CD40* rs1883832 genotypes in ADC patients, **(D)** *CD40* rs1883832 genotypes in stage I and II patients, **(E)** *CD40* rs1883832 genotypes in NSCLC patients with N = 0, **(F)** *CD40* rs1883832 genotypes in NSCLC patients with N = 1. SQ, squamous cell carcinoma; ADC, adenocarcinoma.

Among all NSCLC cases, the *CD40* rs1883832 CT heterozygotes had poorer OS compared to TT and CC homozygotes after 2, 3, and 5 years of observation on univariate analysis (p=0.015, p=0.005, and p=0.017, respectively, **Figure 1**). Prognostic significance for 2-, 3-, and 5-year OS persisted in multivariate analyses adjusted for age, sex, stage, and histological subtypes (p=0.023, p<0.001, and p=0.001, respectively). The same correlation was also observed after stratifying with histological subtype (p=0.075 and p=0.001, respectively). Interestingly, further analysis by stage stratification revealed that the observed correlation was limited in stages I and II (p=0.001), while it disappeared in patients of stages III and IV (p=0.151).

On the contrary, although *BAFFR* rs7290134 (A>G) SNP seemed to be associated with the OS, finally it did not reach the level of statistical significance (p=0.087). In particular, G allele carriers had better 5-year survival compared to AA homozygotes; however, this difference was not statistically significant.

### *CD40* rs1883832 (T>C), *LTβR* rs10849448 (A>G), and *BAFFR* rs7290134 (A>G) Were Associated With Protein Expression

*CD40* rs1883832 was associated with CD40 expression. In particular, *CD40* rs1883832 was associated with total (cytoplasmic and membranous) CD40 expression with CC homozygotes having higher tumorous CD40 (Mann–Whitney U test, p=0.040). In addition, CC homozygotes had higher CD40 expression in stromal cells compared to T allele carriers (Mann–Whitney U test, p=0.036). In addition, *LTβR* rs10849448 SNP was associated with LTβR membranous expression, with AA homozygotes having higher protein levels (p=0.035). Furthermore, the third SNP, *BAFFR* rs7290134 (A>G), was associated with BAFFR membranous expression with A minor allele carriers having lower protein levels (p=0.039).

### *CD40* rs1883832 (T>C) SNP Was Associated With Development of Metastases

*CD40* rs1883832 was associated with development of metastases after the initial assessment. Patients with no development of metastatic disease were mainly CC homozygotes (p=0.022). The same association was also observed in multivariate analysis using age, sex, lymph node infiltration, and histological subtype as cofactors (p=0.019).

## Discussion

During the last years, accumulating evidence supports the role of NF-κB in the pathobiology and management of NSCLC (25). In this context, CD40, BAFFR, and LTβR cell surface receptors, which mainly activate NF-κβ pathways, also seem to be important for NSCLC. In this study, we assessed the clinical value of three SNPs *CD40* (rs1883832), *BAFFR* (rs7290134), and *LTβR* (rs10849448) in NSCLC, with the results further supporting previous findings from our and other groups on the role of these molecules in NSCLC. One of the major findings of the study was the association of *CD40* rs1883832 with NSCLC risk. In line with our observation, Krishnappa et al. reported that the rs1883832 T allele is associated with increased susceptibility to cervical cancer in the Malaysian population (17). Similarly, Shuang et al. have also shown that the rs1883832 T allele is related to sporadic breast cancer risk in Han Chinese women (18). Interestingly, the same allele of rs1883832 (T) has also been documented in a small study (n = 105 cases) and has been correlated with the susceptibility to lung cancer in the Chinese population including not only NSCLC but also SCLC patients (19). Furthermore, TT genotype has also been associated with an increased risk for follicular lymphoma (26).

**Figure 2:**
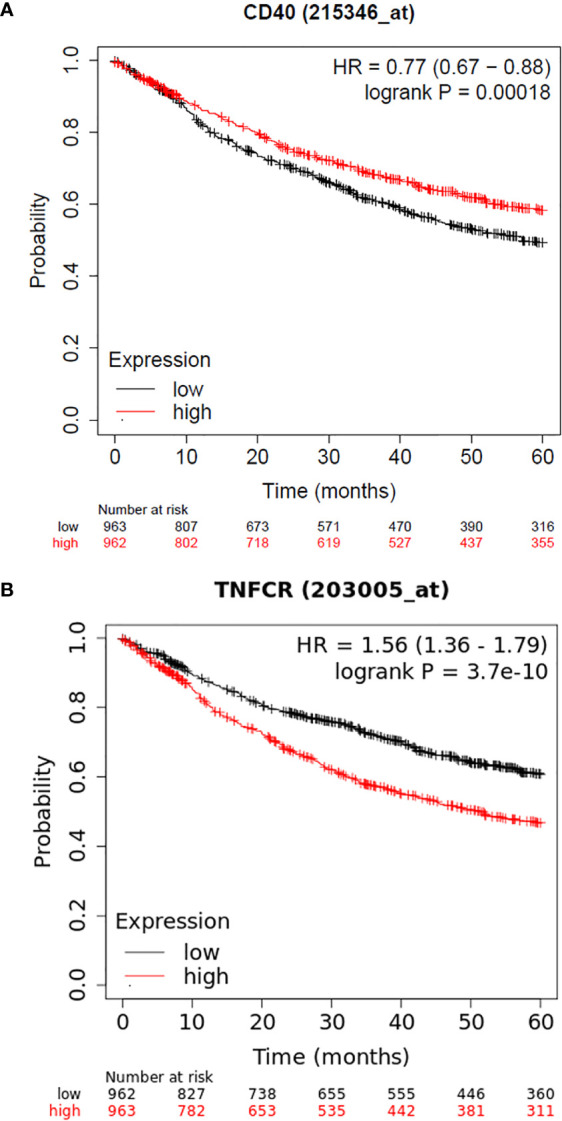
**(A)** Kaplan–Meier curves depicting 5-year OS of NSCLC patients in relation to *CD40* mRNA levels as provided by KM plotter, **(B)** Kaplan–Meier curves depicting 5-year OS of NSCLC patients in relation to *LTβR* mRNA levels as provided by the KM plotter.

Another intriguing finding of the present study was the association of *CD40* rs1883832 with OS. Although the role of this SNP has been studied in many nonmalignant diseases, as mentioned in the introduction, there has not been yet any reported finding on its prognostic significance in solid tumors or hematological malignancies. However, some indirect results are supportive of our observation. Our group has shown that CD40 overexpression in NSCLC patients is associated with improved 5-year OS (6), a finding, which is confirmed by using publicly available data and the KM plotter (**Figure 2A**) (27). In addition, in the current study, we show that *CD40* rs1883832 is associated with total CD40 expression in tumorous cells as well as in stromal cells, suggesting a possible mechanism through which *CD40* rs1883832 may influence survival outcome of NSCLC patients. From a biological point of view, this association could be justified by the genomic position of rs1883832, since it is located on the promoter region and in particular at the -1 base from the start codon of the CD40 and within the Kozak sequence (28). Also supportive is the observation by Skibola et al. according to which follicular lymphoma patients and healthy controls with TT genotypes had decreased plasma circulating soluble CD40 as well as CD40 cell surface expression in dendritic cells from healthy individuals compared to CC homozygotes (26).

Our study also showed that *LTβR* rs10849448 (A>G) SNP is associated with NSCLC risk, with homozygosity for the alternative A allele being related to lower risk for NSCLC, a finding that correlates for the first time this variant with cancer risk. Additionally, our group has reported that *LTβR* expression in NSCLC has prognostic significance (6). The prognostic significance of *LTβR* expression has also been confirmed using the KM plotter and the 203005_at dataset, with higher expression being correlated with poorer outcome (**Figure 2B**). In line with these findings, we also observed that *LTβR* rs10849448 (A>G) SNP was associated with LTβR membranous expression with AA homozygotes having higher protein levels, providing concurrently a potent explanation for the increased risk for NSCLC.

Despite the promising results of our study, we must acknowledge some weak points. A major limitation of our study is that the retrospectively collected samples as well as the size of the cohort did not permit a separate analysis for the discovery and validation subgroups; thus, despite our initial intention to follow a two-phase design, the final analysis was based on the study of the whole cohort. A larger cohort could permit the separate analysis and lead to more robust results. Moreover, molecular profiling of driver mutations and PD-L1 status was not available since the patients were enrolled before immunotherapy era.

## Conclusions

In conclusion, this study shows that two (*CD40* rs1883832 and *LTβR* rs10849448) of the three studied genetic variants are associated with an increased risk for NSCLC, while *CD40* rs1883832 was also significantly associated with OS of patients with NSCLC. However, our findings need to be further validated in another population and more data are needed regarding the functionality of the studied polymorphisms.

## Data Availability Statement

The datasets from sequencing presented in this study can be found in online repository as well as are also available upon request from the corresponding author. The name of the repository and accession number can be found below: European Nucleotide Archive (ENA) (accession number: PRJEB47384).

## Ethics Statement

This study was conducted according to the guidelines of the Declaration of Helsinki and was performed upon approval by the Scientific Committee and the Committee on Research and Ethics of the University Hospital of Patras (Greece, 22/18.2.2015). Written informed consent was obtained from all participants unless the Committee had granted a waiver.

## Author Contributions

Conceptualization, F-ID, AA, AK, and HK. Methodology, AA, AK, F-ID, and VT. Formal analysis, F-ID and AA. Investigation, F-ID, AA, AK, NP, DD, TM, AngK, and HK. Resources, F-ID and HK. Writing—original draft preparation, F-ID, AA, and AK. Writing—review and editing, F-ID, MK, and HK. All authors contributed to the article and approved the submitted version.

## Funding

This research was cofunded by the Hellenic Society of Medical Oncology (HeSMO) through a research funding program as well as from EOGE, an Oncology Research Fund non-profit organization, in Greece.

## Conflict of Interest

The authors declare that the research was conducted in the absence of any commercial or financial relationships that could be construed as a potential conflict of interest.

## Publisher’s Note

All claims expressed in this article are solely those of the authors and do not necessarily represent those of their affiliated organizations, or those of the publisher, the editors and the reviewers. Any product that may be evaluated in this article, or claim that may be made by its manufacturer, is not guaranteed or endorsed by the publisher.
